# Association between Frailty and Hypertension Prevalence, Treatment, and Control in the Elderly Korean Population

**DOI:** 10.1038/s41598-017-07449-5

**Published:** 2017-08-08

**Authors:** Min-gu Kang, Sun-wook Kim, Sol-Ji Yoon, Jung-Yeon Choi, Kwang-il Kim, Cheol-Ho Kim

**Affiliations:** Department of Internal Medicine, Seoul National University College of Medicine, Seoul National University Bundang Hospital, Seongnam, South Korea

## Abstract

Frailty is a common geriatric syndrome characterized by increased risk of disability, hospitalization, and mortality. Hypertension (HTN) is one of the most common chronic medical conditions in the elderly. However, there have been few studies regarding the association between frailty and HTN prevalence, treatment, and control rates. We analyzed data of 4,352 older adults (age ≥ 65 years) from the fifth Korea National Health and Nutrition Examination Survey. We constructed a frailty index based on 42 items and classified participants as robust, pre-frail, or frail. Of the subjects, 2,697 (62.0%) had HTN and 926 (21.3%) had pre-HTN. Regarding frailty status, 721 (16.6%), 1,707 (39.2%), and 1,924 (44.2%) individuals were classified as robust, pre-frail and frail, respectively. HTN prevalence was higher in frail elderly (67.8%) than pre-frail (60.8%) or robust elderly (49.2%) (*P* < 0.001). Among hypertensive patients, frail elderly were more likely to be treated than pre-frail or robust elderly (*P* < 0.001), but the proportion of patients whose blood pressure was under control ( < 150/90 mmHg) was lower in frail elderly (*P* = 0.005). Considering the adverse cardiovascular outcomes associated with frailty, more attention should be paid to the blood pressure control of the frail elderly.

## Introduction

Hypertension (HTN) prevalence increases with aging. HTN is an important risk factor of cerebro-cardiovascular disease, and it has a critical impact on quality of life and maintenance of activities of daily living (ADL) among the elderly. Antihypertensive treatment has been shown to reduce stroke, cardiovascular events, and mortality^1, 2^. However, there remains controversy regarding the optimal treatment of HTN in the elderly, especially the oldest old age group^3^.

Many large-scale trials have proven the necessity of antihypertensive treatment in the elderly. Meta-analyses of large-scale trials for elderly hypertensive patients have revealed significant reductions in cardiovascular morbidity and mortality with antihypertensive treatment^4, 5^. However, an inverse association between blood pressure and cardiovascular risk was found in the oldest age groups among the elderly^6–9^. In particular, the effect of antihypertensive treatment and the ideal blood pressure goal of treatment were not clear in the frail elderly^10^.

Older hypertensive patients are highly heterogeneous, and physiological ability and vulnerability varies widely even for individuals of the same age^11^. Frailty assessments are clinically useful to address the heterogeneity of health status among the elderly. Frailty, as a reflection of decreased physiologic reserve, is closely associated with biological age^12^, concurrent medical conditions, morbidity, and decreased survival in the elderly^13^.

Previous studies suggested that antihypertensive treatment was not safe in the frail elderly^11^. However, other studies showed that antihypertensive treatment has been beneficial both frailer and healthier hypertensive elderly patients. For example, the HYVET (HTN in the Very Elderly Trial) study showed that there was no evidence of an interaction between treatment effect and frailty^14^. Recently, the Systolic Blood Pressure Intervention Trial (SPRINT) study revealed the benefit of strict HTN control even in the frail elderly, with respect to lowering the rate of cardiovascular events and all-cause mortality^15^.

Few studies have examined the effect of frailty on HTN prevalence, treatment, and control in the elderly. Frail elderly are especially vulnerable to cardiovascular disease, and blood pressure control is an effective way to decrease risk of such disease. Therefore, it is important to study the epidemiology of HTN according to frailty status of the elderly people. Analyzing the association between frailty and HTN prevalence, treatment, and control will be helpful for guiding therapeutic decisions for HTN control among the elderly patients.

## Methods

### Study Population

This cross-sectional study was based on data acquired in the fifth Korea National Health and Nutrition Examination Survey (KNHANES V), conducted from 2010 to 2012. These surveys have been conducted periodically since 1998 to assess the health and nutritional status of Koreans, monitor trends in health risk factors and the prevalence of major chronic diseases, and provide data for the development and evaluation of health policies and programs in Korea^16^.

Twenty households were selected throughout 192 regions for each of the years, and 10,000 individuals aged 1 year and over were targeted for KNHANES. To reduce the limitation of seasonal variations, KNHANES V was conducted continuously year round. The KNHANES has been conducted by the Korean Centers for Disease Control & Prevention since 2007. There were 4,557 older adults (age ≥ 65) in the KNHANES V. After excluding 10 participants with missing blood pressure data, and 195 participants with missing questionnaire data about HTN, 4,352 participants were included in the study. This study was approved by the Institutional Review Board of Seoul National University Bundang Hospital (IRB No. B-1610-365-101), which waived the requirement for informed consent. In addition to this, all methods were performed in accordance with the Strengthening the Reporting of Observational Studies in Epidemiology Statement and regulation of the institutional review board.

### Subject Evaluation and Laboratory Study

Blood pressure was measured on the right arm, by trained nurses, using a mercury sphygmomanometer (Baumanometer® Desk model 0320, Baum, USA) with an appropriately sized cuff after participants remained still for at least 5 minutes in a sitting posture. During the measurement period, participants were seated leaning against the back of a chair, while their feet remained flat on the floor. The right arm of participant was located at the middle of the cuff to be at the level of the heart. Blood pressure was measured 3 times, and the mean blood pressure of the second and third measured value was used to determine the final systolic and diastolic blood pressure.

Blood samples were collected from each participant during the survey. Body mass index (BMI) was calculated by dividing body weight (kg) by height^2^ (m^2^). Waist circumference was measured at the narrowest point from the lower border of the rib cage to the iliac crest. Information about household income, level of education, and life style factors was derived from a self–reported questionnaire. A nutrition survey was conducted by the survey team via interview.

### Definitions

HTN was defined as blood pressure ≥ 140/90 mmHg or taking antihypertensive medications. Pre-HTN was defined as 120 mmHg ≤ systolic blood pressure < 140 mmHg and 80 mmHg ≤ diastolic blood pressure < 90 mmHg. HTN treatment was defined if patients took antihypertensive medication 20 days or more in a month. We analyzed our data with the blood pressure target of 150/90 mmHg according to current guidelines^17, 18^.

Diabetes was defined as fasting blood sugar ≥ 7 mmol/L (126 mg/dL), taking an oral hypoglycemic agent or insulin, or diagnosed with diabetes by a medical doctor.

Glomerular filtration rate (GFR) was calculated by using the Chronic Kidney Disease Epidemiology Collaboration (CKD-EPI) equation^19^. Chronic kidney disease (CKD) was defined as estimated GFR < 60 mL/min per 1.73 m^2^.

Smokers were defined as those who smoked 5 packs of cigarettes in a lifetime or more, or they were current smokers. Exercise was defined as either 3 times or more per week of 20 minutes or longer strenuous physical activity, 5 times or more per week of 30 minutes or longer moderate physical activity or walking exercise.

### Frailty index

We developed the frailty index using a cumulative deficit model, including symptoms, signs, abnormal laboratory values, disease status, and disabilities^20^. The frailty index, calculated as a ratio of deficits present out of the total number of possible deficits, gives a continuous score from total fitness (0) to total frailty (1). This means that the more deficits an individual has, the frailer they will be^21^.

Our frailty index consists of 42 items from the KNHANES V. The items used to calculate the index value included comorbidities, functional abilities, signs and symptoms, and laboratory values. Comorbidities included bronchial asthma, chronic obstructive pulmonary disease (COPD), diabetes, dyslipidemia, cataract, cardiovascular disease, stroke, arthritis, anemia, cancer, and depression. Functional abilities consisted of inactivity, exercise capacity, ADL limitation, social activity limitation, self-care ability, difficulty in chewing hard foods, hearing impairment, and anosmia. Signs and symptoms consisted of pain or discomfort, back pain, weight loss, dyspnea, depression or anxiety, fatigue, suicidal ideation, and stress. Laboratory values consisted of systolic blood pressure, diastolic blood pressure, heart rate regularity, pulmonary function test, hemoglobin, blood urea nitrogen, creatinine, vitamin D, total cholesterol, triglyceride, high density lipoprotein (HDL)-cholesterol, fasting glucose, and urine protein. Additional items were current smoking and obesity.

We classified the study participants as robust (frailty index ≤ 0.10), pre-frail (0.10 < frailty index ≤ 0.21), or frail (frailty index > 0.21) according to previous criteria^15, 21^.

### Statistical analysis

All statistical analyses were performed using the SPSS version 19.0 statistical package (SPSS Inc., Chicago, IL). Continuous variables were expressed as mean ± standard deviation, and were compared by either the unpaired Student’s *t*-test or one-way analysis of variance (One-way ANOVA). Discrete variables were expressed as counts and percentages, and the proportions were compared by using the Chi-square test. We used binary logistic regression analysis to estimate the odds ratios (OR) and 95% confidence intervals (CI), adjusting for factors that were considered to potentially influence the results. All statistical analyses were two-tailed, and P-values < 0.05 were taken as statistically significant.

### Data availability

All data are available at https://knhanes.cdc.go.kr/.

## Results

### General characteristics of the study population

**Figure 1:**
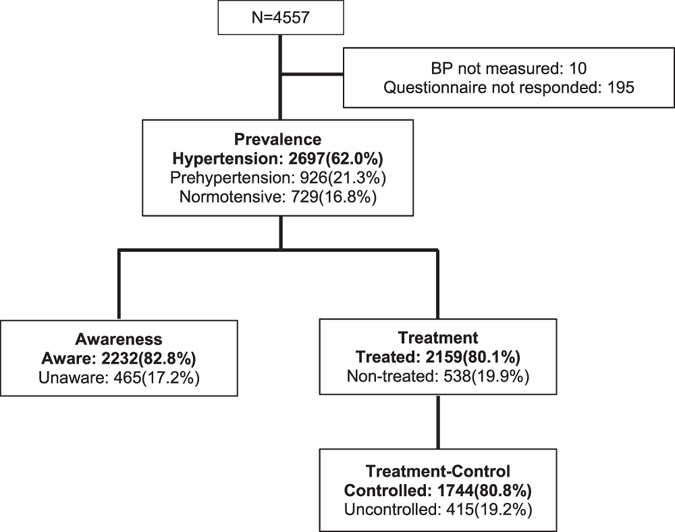
HTN prevalence, awareness, treatment, and control rate in Korean elderly. 62.0% elderly have HTN, and 80.1% hypertensive elderly were treated; treatment-control rate was 80.8%.

The mean age of the study population was 72.6 ± 5.4 years and 1,855 (42.6%) participants were men. Of all subjects, 2,697 (62.0%) had HTN and 926 (21.3%) had pre-HTN. Among hypertensive patients, 2,159 patients (80.1% of the HTN patients) were treated. Among treated hypertensive patients, the blood pressure of 1,744 patients (80.8% of the treated HTN patients) was controlled under 150/90 mmHg. (Fig. 1) In terms of frailty status, 721 (16.6%), 1,707 (39.2%), and 1,924 (44.2%) patients were classified as robust, pre-frail, and frail, respectively. General characteristics related to frailty status are presented in Table 1.

**Table 1:** General characteristics of the study population according to frailty status (robust, pre-frail, frail).

	Robust (N = 721)	Pre-frail (N = 1707)	Frail (N = 1924)	*P-value*
Age	71.6 (4.9)	72.1 (5.3)	73.5 (5.5)	<0.001
SBP (mmHg)	127.3 (15.5)	129.9 (17.6)	130.4 (18.2)	<0.001
DBP (mmHg)	74.0 (8.6)	73.9 (9.7)	73.1 (10.6)	0.026
PP (mmHg)	53.3 (13.3)	56.0 (14.9)	57.3 (15.9)	<0.001
Waist circumference (cm)	82.7 (7.9)	83.4 (9.1)	84.6 (10.0)	<0.001
BMI (kg/m^2^)	23.2 (2.5)	23.6 (3.1)	24.2 (3.6)	<0.001
Hb (g/dl)	14.2 (1.3)	13.8 (1.4)	13.4 (1.5)	<0.001
GFR (60 ml/min/1.73 m^2^)	80.3 (10.3)	78.3 (12.6)	75.1 (16.0)	<0.001
Sodium intake (g/d)	4.7 (3.2)	4.1 (2.9)	3.7 (2.8)	<0.001
Sex (male sex)	421 (58.4%)	813 (47.6%)	621 (32.3%)	<0.001
Income quartile (low/mid-low/mid-high/high)	38.5%/29.5%/19.5%/12.5%	47.4%/26.6%/14.5%/11.6%	60.7%/22.2%/9.6%/7.4%	<0.001
Level of education (1^st^/2^nd^/3^rd^/4^th^)	47.6%/13.7%/22.9%/15.8%	61.0%/14.2%/16.6%/8.2%	77.6%/9.9%/9.6%/2.9%	<0.001
Smoking	48 (7.0%)	230 (13.8%)	261 (13.7%)	<0.001
Exercise	474 (69.0%)	780 (46.9%)	644 (34.1%)	<0.001
HTN	355 (49.2%)	1038 (60.8%)	1304 (67.8%)	<0.001
HTN treatment	258 (72.7%)	810 (78.0%)	1091 (83.7%)	<0.001
Diabetes	59 (9.6%)	282 (18.9%)	465 (28.9%)	<0.001
Dyslipidemia	168 (29.4%)	734 (51.5%)	951 (60.8%)	<0.001
Stroke	11 (1.5%)	62 (3.6%)	161 (8.4%)	<0.001
MI, angina	18 (2.6%)	113 (6.7%)	210 (11.0%)	<0.001
CKD	16 (2.5%)	138 (8.9%)	311 (18.7%)	<0.001

SBP: systolic blood pressure, DBP: diastolic blood pressure, PP: pulse pressure, BMI: body mass index, Hb: hemoglobin, GFR: glomerular filtration rate, HTN: hypertension, MI: myocardial infarction, CKD: chronic kidney disease.

Income quartile: household income/month, Low: household income/month < 650 US dollars, Mid-low: 650 US dollars ≤ household income/month < 1300 US dollars, Mid-high: 1300 US dollars ≤ household income/month < 2100 US dollars, High: household income/month ≥ 2100 US dollars.

Level of education: 1^st^: elementary school or lower, 2^nd^: middle school, 3^rd^: high school, 4^th^: college or higher.

Dyslipidemia: either hypercholesterolemia, hypertriglyceridemia, low HDL-cholesterolemia, or a combination. (Hypercholesterolemia: 12 h-fasting total cholesterol ≥ 6.22 mmol/L (240 mg/dL) or taking hypercholesterolemia medications, Hypertriglyceridemia: 12 h-fasting triglyceride ≥ 2.27 mmol/L (200 mg/dL), Low HDL-cholesterolemia: 12 h-fasting HDL-cholesterol < 1.04 mmol/L (40 mg/dL)).

### Comparison of HTN prevalence and treatment, and control rate according to frailty status

**Figure 2:**
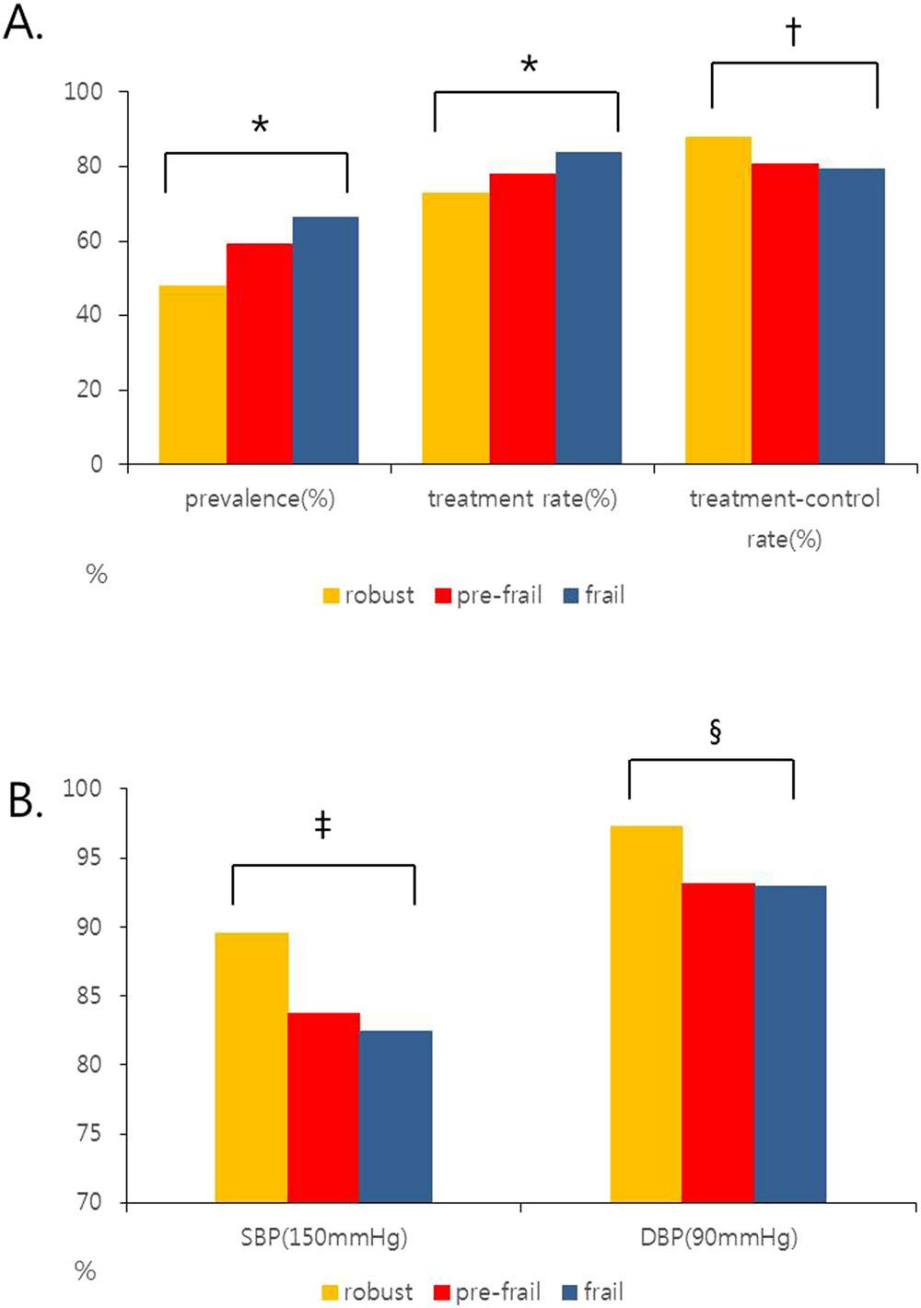
(**A**) Comparison of HTN prevalence and treatment, and control rate according to frailty status. (*P value < *0.001*, ^†^ *P value* = *0.005*). HTN prevalence was higher in frail elderly than pre-frail or robust elderly. (*P* < *0.001*) In hypertensive patients, frail elderly are more likely to be treated than pre-frail or robust elderly (*P* < *0.001*). In treated patients, the proportion of patients whose blood pressure was controlled under 150/90 mmHg was significantly lower in frail elderly (*P* = *0.005*). (**B**) Comparison of SBP and DBP control rate in treated patients according to frailty status. (^‡^ *P value = 0.020*, ^§^ *P value* = *0.032*). SBP: systolic blood pressure, DBP: diastolic blood pressure. In treated patients, the proportion of patients whose systolic blood pressure was controlled under 150 mmHg was significantly lower in frail elderly (*P* = *0.020*), and the proportion of patients whose diastolic blood pressure was controlled under 90 mmHg was also lower in frail elderly (*P* = *0.032*).

**Figure 3:**
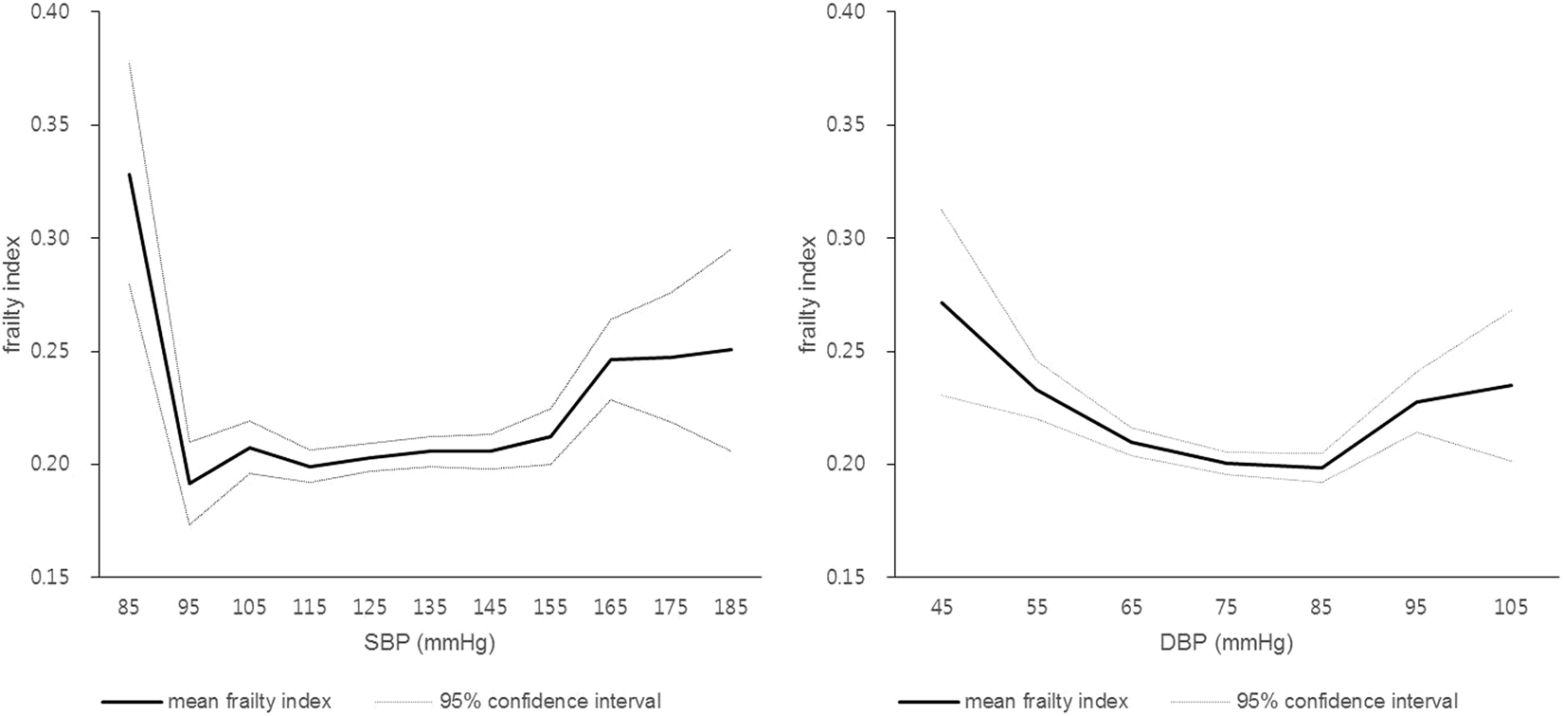
Frailty index (mean) in Korean elderly according to SBP and DBP. SBP: systolic blood pressure, DBP: diastolic blood pressure. U-shape relationship between frailty index and blood pressure was observed.

HTN prevalence was higher in frail elderly (67.8%) than pre-frail (60.8%) or robust elderly (49.2%) (*P* < *0.001*). Among hypertensive patients, frail elderly are more likely to be treated than pre-frail or robust elderly (*P* < *0.001*). However, the proportion of patients whose blood pressure was controlled under 150/90 mmHg was lower in frail elderly (*P* = *0.005*) (Fig. 2A). Among treated patients, the proportion of patients whose systolic blood pressure was controlled under 150 mmHg was lower in frail elderly (*P* = *0.020*), and the proportion of patients whose diastolic blood pressure was controlled under 90 mmHg was also lower in frail elderly (*P* = *0.032*) (Fig. 2B). There was no significant difference in systolic blood pressure according to frailty status in treated patients (frail: 133.1 ± 17.4 mmHg, pre-frail: 133.4 ± 17.0 mmHg, robust: 131.3 ± 13.0 mmHg, *P* = 0.204), however, diastolic blood pressure was lower in frail patients (73.0 ± 10.6 mmHg) than pre-frail (74.2 ± 9.6 mmHg) or robust (74.1 ± 8.1 mmHg) patients (*P* = 0.019). When we analyzed the association between frailty index and systolic or diastolic blood pressure of the entire study population, there was U-shape relationship between them. In other words, the mean frailty index was higher in patients with high or low blood pressure (Fig. 3).

### Factors associated with blood pressure control

To identify the factors associated with poor blood pressure control, comparison between controlled and uncontrolled groups was performed. Frailty index, and female sex were associated with poor control of HTN in a univariate analysis (Table 2). In a binary logistic regression analysis, frailty status (odds ratio: 2.077 [pre-frail], 2.536 [frail]; 95% CI: 1.242–3.475 [pre-frail], 1.506–4.272 [frail]; *P* = *0.005* [pre-frail], *P* < *0.001* [frail]) was significantly related to poor blood pressure control (Table 3). Additionally, we tried to show the effect of uncontrolled blood pressure on the frailty status. In a multiple logistic regression analysis, uncontrolled blood pressure was significantly associated with frailty status (odds ratio: 2.044 [pre-frail], 2.326 [frail]; 95% CI: 1.234–3.384 [pre-frail], 1.396–3.874 [frail]; *P* = *0.005* [pre-frail], *P* = *0.001* [frail]) (Table 4).

**Table 2:** Univariate analysis: factors associated with blood pressure control (target blood pressure: 150/90 mmHg)

	Target blood pressure (150/90 mmHg)		
	Controlled (N = 1744)	Uncontrolled (N = 415)	*P-value*
Age	72.9 (5.2)	73.2 (5.9)	0.311
Sex (male sex)	672 (38.5%)	133 (32.0%)	0.014
SBP (mmHg)	127.3 (11.9)	156.9 (12.6)	<0.001
DBP (mmHg)	71.5 (8.4)	82.0 (11.5)	<0.001
PP (mmHg)	55.8 (11.9)	74.9 (18.3)	<0.001
Waist circumference (cm)	86.0 (9.0)	85.1 (9.5)	0.092
BMI (kg/m^2^)	24.6 (3.3)	24.7 (3.4)	0.610
Hb (g/dl)	13.6 (1.5)	13.5 (1.5)	0.700
GFR (60 ml/min/1.73 m^2^)	75.3 (14.6)	73.8 (16.6)	0.116
Sodium intake (g/d)	3.8(2.8)	3.7(2.6)	0.491
Frailty index	0.22 (0.10)	0.24 (0.11)	<0.001
Income quartile (low/mid-low/mid-high/high)	51.5%/25.8%/13.1%/9.6%	53.3%/24.2%/12.3%/10.1%	0.856
Level of education (1^st^/2^nd^/3^rd^/4^th^)	66.7%/12.2%/14.9%/6.2%	70.1%/10.2%/14.1%/5.6%	0.561
Smoking	176 (10.2%)	30 (7.3%)	0.073
Exercise	766 (44.5%)	170 (41.4%)	0.252
Diabetes	428 (28.4%)	105 (29.8%)	0.683
Dyslipidemia	839 (57.8%)	194 (57.6%)	0.932
Stroke	143 (8.2%)	33 (8.0%)	0.868
MI, angina	167 (9.6%)	49 (11.8%)	0.173
CKD	251 (16.2%)	71 (19.5%)	0.126

SBP: systolic blood pressure, DBP: diastolic blood pressure, PP: pulse pressure, BMI: body mass index, Hb: hemoglobin, GFR: glomerular filtration rate, MI: myocardial infarction, CKD: chronic kidney disease.

Income quartile: household income/month, Low: household income/month < 650 US dollars, Mid-low: 650 US dollars ≤ household income/month < 1300 US dollars, Mid-high: 1300 US dollars ≤ household income/month < 2100 US dollars, High: household income/month ≥ 2100 US dollars.

Level of education: 1^st^: elementary school or lower, 2^nd^: middle school, 3^rd^: high school, 4^th^: college or higher.

Dyslipidemia: either hypercholesterolemia, hypertriglyceridemia, low HDL-cholesterolemia, or a combination. (Hypercholesterolemia: 12 h-fasting total cholesterol ≥ 6.22 mmol/L (240 mg/dL) or taking hypercholesterolemia medications, Hypertriglyceridemia: 12 h-fasting triglyceride ≥ 2.27 mmol/L (200 mg/dL), Low HDL-cholesterolemia: 12 h-fasting HDL-cholesterol < 1.04 mmol/L (40 mg/dL)).

**Table 3:** Binary logistic regression analysis: factors associated with blood pressure control (target blood pressure: 150/90 mmHg).

	Target blood pressure (150/90 mmHg)		
	*P-value*	Exp (β)	95% Confidence interval
Age	0.697	0.995	0.969–1.021
BMI (kg/m^2^)	0.710	0.992	0.953–1.033
Sodium intake (g/d)	0.920	1.000	1.000–1.000
Female sex	0.775	1.042	0.784–1.386
Smoking	0.222	0.739	0.455–1.201
Exercise	0.594	0.932	0.719–1.208
Diabetes	0.552		
IGT	0.286	1.181	0.870–1.604
Diabetes	0.530	1.100	0.817–1.483
Dyslipidemia	0.240	0.856	0.661–1.109
CKD	0.383	1.159	0.832–1.613
Frailty status	0.002		
Pre-frail	0.005	2.077	1.242–3.475
Frail	<0.001	2.536	1.506–4.272

BMI: body mass index, IGT: impaired glucose tolerance (5.4 mmol/L (100 mg/dL) ≤ fasting blood sugar ≤ 6.9 mmol/L (125 mg/dL)), CKD: chronic kidney disease, Dyslipidemia: either hypercholesterolemia, hypertriglyceridemia, low HDL-cholesterolemia, or a combination. (Hypercholesterolemia: 12 h-fasting total cholesterol ≥ 6.22 mmol/L (240 mg/dL) or taking hypercholesterolemia medications, Hypertriglyceridemia: 12 h-fasting triglyceride ≥ 2.27 mmol/L (200 mg/dL), Low HDL-cholesterolemia: 12 h-fasting HDL-cholesterol < 1.04 mmol/L (40 mg/dL)).

**Table 4:** Multiple logistic regression analysis: factors associated with frailty status.

	Pre-frail			Frail		
	*P-value*	Exp (β)	95% **C**onfidence interval	*P-value*	Exp (β)	95% **C**onfidence interval
Age	0.980	1.000	0.964–1.036	0.006	1.052	1.015–1.091
BMI (kg/m^2^)	0.460	1.022	0.965–1.082	0.001	1.106	1.043–1.172
Female sex	<0.001	2.150	1.533–3.015	<0.001	4.180	2.936–5.953
Smoking	0.016	2.246	1.164–4.334	<0.001	3.856	1.973–7.536
Exercise	<0.001	0.515	0.366–0.725	<0.001	0.286	0.202–0.406
Diabetes	0.211	1.139	0.929–1.397	0.001	1.413	1.148–1.739
Dyslipidemia	<0.001	2.379	1.694–3.342	<0.001	3.607	2.544–5.114
CKD	0.001	3.867	1.803–8.295	<0.001	7.643	3.583–16.303
Uncontrolled HTN*	0.005	2.044	1.234–3.384	0.001	2.326	1.396–3.874

BMI: body mass index, CKD: chronic kidney disease, Dyslipidemia: either hypercholesterolemia, hypertriglyceridemia, low HDL-cholesterolemia, or a combination. (Hypercholesterolemia: 12 h-fasting total cholesterol ≥ 6.22 mmol/L (240 mg/dL) or taking hypercholesterolemia medications, Hypertriglyceridemia: 12 h-fasting triglyceride ≥ 2.27 mmol/L (200 mg/dL), Low HDL-cholesterolemia: 12 h-fasting HDL-cholesterol < 1.04 mmol/L (40 mg/dL)), Uncontrolled HTN*: target blood pressure: 150/90 mmHg.

## Discussion

In this study, we showed that HTN prevalence and treatment rate was higher, but control rate was lower among the frail elderly people. Furthermore, frailty status was an independent factor associated with poor blood pressure control. In addition, uncontrolled blood pressure was associated with pre-frail or frail status of the study population. Interestingly, the mean frailty index was greater in elderly patients who had high or low systolic and diastolic blood pressure. Considering the significance of frailty on future cardiovascular events and mortality, more attention should be paid to frail hypertensive patients for the better management of HTN and improvement of prognosis.

Frailty statuses (robust, pre-frail, and frail) were meaningfully associated with prevalence of HTN. The association has been published in the previous studies^22–24^. Maybe the reason for the association is that frailty is a condition associated with problems across multiple physiological systems. HTN treatment rate was significantly higher among the frail elderly. Because frailty is associated with comorbidity, frail elderly tend to use medical treatment more frequently, and as accessibility to healthcare institutions has increased, the HTN treatment rate may be increasing accordingly.

But we observed that HTN control rate was low in frail elderly. There were several reasons that may explain why the control rate was low in frail elderly. First, the target level for blood pressure control has been unclear in frail elderly, and, until recently, clinicians considered individual tolerability in setting up treatment regimens. Second, isolated systolic HTN is common in elderly hypertensive patients. In such cases, many clinicians are concerned that strict systolic blood pressure control using antihypertensive drugs might cause excessively low diastolic blood pressure. Too low diastolic blood pressure might increase the risk of adverse events, such as a stroke^25^, an injurious fall or syncope potentially. As a result, clinicians have not consistently and strictly controlled blood pressure of frail elderly. Third, frailty was associated with an unhealthy lifestyle. In this study, frail elderly had high BMI, large waist circumference, high smoking rate, and low physical activity rate. High BMI and large waist circumference are associated with poor dietary habits. Because lifestyle modification is also important in blood pressure control, it is difficult to treat HTN in frail elderly. Lastly, we have to consider comorbidities such as diabetes, dyslipidemia, renal impairment, stroke, and cardiovascular disease in frail elderly. It is possible that their general physical condition and polypharmacy had a considerable influence on the effects of antihypertensive drugs.

Although, there have been concerns regarding strict blood pressure control for frail older adults, but SPRINT study revealed the benefit of strict HTN control for the patients. There was a significantly lower rate of cardiovascular events and all-cause mortality in the intensive treatment group whose systolic blood pressure was controlled less than 120 mmHg, even the frail elderly^15^. Additionally, the overall rate of serious adverse events and absolute rates of adverse events, such as hypotension, syncope, electrolyte abnormalities, acute kidney injury, and injurious fall, were not significantly different between treatment groups^15^. Uncontrolled HTN is the cause of serious cardiovascular events. In addition, previous studies showed that HTN is associated with future ADL/IADL limitation or disability even in patients without stroke^26, 27^. Blood pressure control helps prevent the occurrence of additional morbidity and consequently helps maintain the current quality of life and ADL/IADL of elderly people.

In this study, frail elderly had lower blood pressure control rate. Additionally, there was a U-shaped relationship between systolic or diastolic blood pressure and frailty index. In other words, frail elderly people consist of two distinct groups, people with high blood pressure and low blood pressure. Accordingly, for the better control of blood pressure of the frail elderly people, we should pay attention to the frail elderly people who have high blood pressure.

For the frail elderly, blood pressure monitoring and drug side effect confirmation are required through additional early visits to confirm tolerability, when we start a new HTN drug. Once these assessments confirm the intolerance of HTN medication, individualized management, such as drug modification and dose titration, will be required. Especially proper management is necessary to prevent falls when a new HTN drug is started in elderly, because antihypertensive drug initiation during the first 45 days of treatment was associated with an increased risk of falls in community-dwelling elderly^28^. This study has strengths and limitations. First, the study population was drawn from nationally representative samples of older adults in Korea. Therefore, the results of this study are easily generalizable. Second, statistically significant results were attained after adjusting for multiple covariates. Limitations of this study are as follows. First, we cannot know the causal and temporal relationship between frailty and HTN because of limitations of cross-sectional study design. Second, data from the KNHANES V were based on self-reported questionnaires, which present the limitation of recall bias. Third, KNHANES V contains questionnaires about the use of HTN medication and the number of doses per month, but does not include questionnaires about the type of HTN medication and the number of concomitant use. In conclusion, considering frailty status is an independent risk factor for poor blood pressure control, more attention should be paid to control blood pressure in frail elderly.
